# Luteolin: A Flavonoid that Has Multiple Cardio-Protective Effects and Its Molecular Mechanisms

**DOI:** 10.3389/fphar.2017.00692

**Published:** 2017-10-06

**Authors:** Yuanyuan Luo, Pingping Shang, Dongye Li

**Affiliations:** ^1^The First Clinical College, Nanjing University of Chinese Medicine, Nanjing, China; ^2^The Affiliated Hospital of Xuzhou Medical University, Xuzhou, China; ^3^Institute of Cardiovascular Disease Research, Xuzhou Medical University, Xuzhou, China

**Keywords:** luteolin, cardio-protection, target effectors, I/R injury, heart failure, atherosclerosis

## Abstract

Cardiovascular disease (CVD) has become the leading cause of morbidity and mortality worldwide. A well-monitored diet with a sufficient intake of fruits and vegetables has been confirmed as a primary prevention of CVD. Plant constituents such as flavonoids have been shown to confer healthy benefits. Luteolin (Lut), a kind of flavonoid, possesses anti-oxidative, anti-tumor, and anti-inflammatory properties. Recent scientific literature has reported the cardiac protective effects of Lut *in vitro* and *in vivo*. Therefore, the aim of this review is to provide an update and detailed overview with cardio-protective molecular mechanisms of Lut with a focus on multiple intrinsic and extrinsic effectors. We further explore how these mechanisms participate in ischemia/reperfusion (I/R) injury, heart failure (HF) and atherosclerosis (AS). A proper understanding of the cardiovascular protective effects and the relative mechanisms of Lut may provide the possibility of new drug design and development for CVD. With the previous studies mainly focused on basic research, we need to advance the prospects of its further clinical utilization against CVD, large prospective clinical trials of Lut are needed to observe its therapeutic effects on patients with I/R injury, HF and AS, especially on the effective therapeutic dosage, and safety of long-term administration.

## Introduction

Flavonoids are naturally occurring compounds that are universally present in the plant kingdom (Hertog et al., 1992). The basic structure of flavonoids consists of an aromatic A-ring and a heterocyclic C-ring, which are connected with an aromatic B-ring through a carbon–carbon bridge. Based on heterocyclic C-ring variation, flavonoids are divided into six subclasses: flavones, flavonols, flavanones, catechins, or flavanols, anthocyanidins and isoflavones (Weng and Yen, 2012). Data sets from the Australian National Nutrition Survey (10,851 subjects>18 years) on flavonoid content of foods showed that the key sources of flavonoids included tea (black and green tea), vegetable (onion, celery, parsley, broccoli, English spinach, bean), fruits (apple, grape, coffee, oranges, lemon, mandarin, cherry, blueberry) and wine (Somerset and Johannot, 2008). Since discovery of flavonoids in 1936, more than 5,000 structures of flavonoids have been reported with a wide range of molecular diversity. The Women’s Health Study with 38,408females ≥ 45years showed that the median intake of total flavonoids ranging from 8.88 to 47.44 mg/day in 11.5 years follow-up (Wang et al., 2009). The Dutch National Food Consumption Survey indicated that people in Netherlands have an average mixed flavonoids intake of 23 mg/day. Epidemiological evidence shows that intake of these compounds does not only inhibit tumor formation and inflammation, but also effectively prevents cardiovascular diseases (CVD). In the Zutphen Elderly Study, Hertog et al. (1993) reported that the intake of flavonoids was inversely related to mortality (*p* = 0.015) and myocardial infarction (*p* = 0.08). Schüssler et al. (1995) reported that main flavonoids from Crataegus species increased coronary flow and relaxation velocity Langendorff heart models. The greatest increases of coronary flow (186%) and relaxation velocity (104%) were found in the luteolin-7-glucoside group (Schüssler et al., 1995). Therefore, generous dietary intake of flavonoids may be an effective primary prevention of CVD.

**FIGURE 1:**
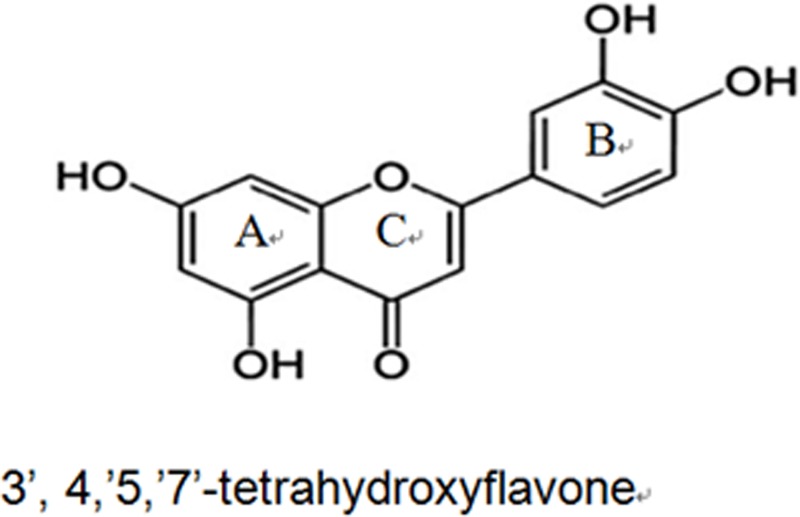
The basic structure of Lut consists of an aromatic A-ring and a heterocyclic C-ring, which are connected with an aromatic B-ring through a carbon-carbon bridge.

Luteolin (3′, 4′, 5′, 7′-tetrahydroxyflavone, Lut) is one of the most prevalent flavones. Molecular formula of Lut is C_15_H_10_O_6_ and the structure is showed in **Figure 1** (Weng and Yen, 2012). A variety of vegetables, fruits and herbs such as carrot, cabbage, artichoke, tea, celery and apple are rich in Lut. The median intake of Lut is 0.01–0.20 mg/day (0.0349–0.698 μmol/day) (Wang et al., 2009). Lut is absorbed by the intestinal mucosa. After oral administration of 14.3 mg/kg Lut, the maximum plasma concentration (Cmax) was 1.97 ± 0.15g/ml. The time to reach Cmax was 1.02 ± 0.22 h and the half-life of Lut (t1/2) was 4.94 ± 1.2 h. It was converted to free Lut, glucuronide and sulfate-conjugates of Lut and of *O*-methyl Lut (diosmetin or chrysoeriol) in the plasma of rats. Free Lut is also presentin human plasma (Lopez-Lazaro, 2009). Through intrinsic and extrinsic signaling pathways, Lut is an active compound with anti-oxidant, anti-tumor, anti-inflammatory, and anti-apoptotic activities (Sun et al., 2015; Zhang et al., 2016). Due to its potent anti-tumor and anti-inflammatory flavonoid, earlier studies and clinical trials on Lut had thus focused on cancer and inflammation. Since the 1950s to date, there has been an increase in the number of reports on the cardiovascular effects of Lut. Marijan-Jankovic (1957), N reported that Lut has effects on the heart and vascular vessels. With current advancements in the understanding of oxidative stress and inflammatory mechanisms of the cardiovascular system, Lut exhibits strong cardiovascular protective activities via complex signal transduction pathways and target effectors. Indeed, high dietary intake of Lut is related to a decreased risk of acute myocardial infarction (Qi et al., 2011; Si et al., 2014). The cardio protection mainly stems from decreased myocardial apoptosis, diminished myocardial infarct size and through enhanced left ventricular ejection fraction (Liao et al., 2011).

It has been proposed that Lut exerts protective effects in a multitude of CVD, including coronary artery disease (CAD), heart failure (HF), and atherosclerosis (AS). Relevant studies on this topic have mainly focused on the target effectors of Lut using *in vitro* and *in vivo* models. Here we present an update and detailed overview of the molecular mechanisms that orchestrate in cardiac protection conferred by Lut, mainly on the anti-I/R injury, cardiac function in HF and mechanism of anti-AS.

### Anti-I/R Injury Mechanism of Lut in CAD

**FIGURE 2:**
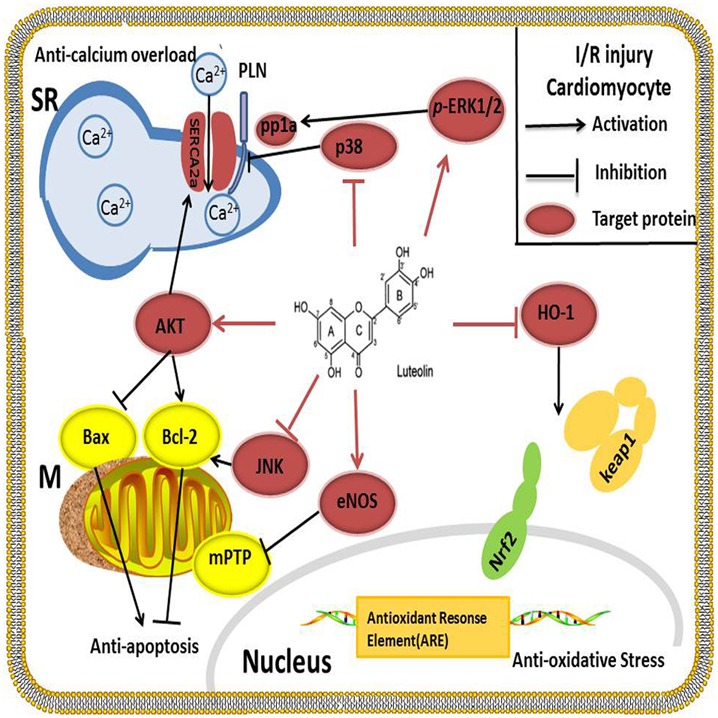
Lut alleviates I/R injury by inhibiting apoptosis by up-regulating AKT, increasing BCL-2 protein and decreasing BAX protein. SERCA2a is the key protein involved in Ca^2+^ uptake from the cytosol into the SR. SERCA2a activity is improved after activation of the PI3K/AKT signaling pathway; at the same time, Lut works as a p38 MAPK pathway inhibitor to suppress the phosphorylation of PLN, thereby enhancing the activity of SERCA2a and reducing the Ca^2+^ overload. Oxidative stress is blocked by Lut through HO-1, which enhances the binding of Nrf2 to the ARE. Lut inhibits JUK and increases p-ERK1/2 to enhance the contraction of cardiomyocytes. Lut up-regulates the myocardial eNOS pathway and inhibits the mitochondrial permeability transition pore to protect the heart against I/R injury.

Coronary artery disease is a serious problem affecting human health (Moran et al., 2013). The most efficient method used to reduce mortality in these patients is to reperfuse the myocardium by interventional treatment or pharmacological therapy (Cabadés et al., 2010). However, myocardial ischemia/reperfusion (I/R) injury followed by reperfusion can damage the myocardium, inducting reperfusion arrhythmia, no-reflow phenomenon, and systolic/diastolic dysfunction, which affects the outcomes of reperfusion therapy. Therefore, many researchers are seeking more effective drugs that can treat I/R injury in CAD patients. Emerging evidence has revealed that Lut regulates vital effectors involved in the process of I/R injury and has displayed satisfactory therapeutic value (**Figure 2**).

#### Lut Activates Anti-Apoptosis Key Protein AKT

The phosphoinositide 3-kinase (PI3K)/AKT pathway is a key pathway mediating in cell growth, metabolism and survival (Brazil et al., 2004). AKT has also been confirmed to regulate the apoptotic pathological process of I/R injury (Ha et al., 2008). In cardiac-protection of Lut against I/R injury, AKT is widely and thoroughly studied: First, Lut suppresses apoptosis by up-regulating AKT in a simulated ischemia/reperfusion (sI/R) model. Pretreatment with Lut significantly inhibited apoptosis, increased BCL-2 expression and the BCL-2/BAX ratio as well as decreased the expression of BAX. Moreover, PI3K inhibitor LY294002 which downregulates AKT expression markedly diminished the function of Lut-induced positive contraction and attenuated the Lut protection against apoptosis in sI/R cardiomyocytes (Fang et al., 2011). Second, previous studies have shown that Ca^2+^ overload is another major mechanism of myocardial I/R injury. Sarcoplasmic reticulum (SR) Ca^2+^-ATPase (SERCA2a) is a key protein involved in Ca^2+^ uptake from the cytosol into the SR and its dysfunction impairs myocardial systolic/diastolic function (Pinton et al., 2008). Our previous study found that Lut improved SERCA2a activity partially through activation of the PI3K/AKT signaling pathway and increased the phosphorylation of AKT (p-Akt) at amino acids 308 and 473 levels, but did not change the SERCA2a expression at protein levels. The infarct size, pro-apoptosis proteins, and lactate dehydrogenase release (LDH) release were reduced by Lut during I/R injury *in vivo* (Nai et al., 2015), and the same effects were seen in diabetic rats (Sun D. et al., 2012).

#### Lut Blocks Oxidative Stress through HO-1

Heme oxygenase-1 (HO-1) is an integral membrane protein located in the smooth endoplasmic reticulum. It is under oxidant-responsive transcription factors NF-E2-related factor 2 (Nrf2) regulation, a pathway that promotes cytoprotection against oxidative stress. When Kelch-like ECH-associated protein 1(Keap1) binds Nrf2, transcriptional activation of Nrf2 is repressed. After exposure to oxidative stress, Nrf2 is released from Keap1 and translocates into nuclei, binding to the antioxidant responsive elements (ARE), which in turn activates Nrf2 target gene HO-1 (Motohashi and Yamamoto, 2004). It appears, therefore, that blocking the oxidative stress through the HO-1 is another possible cytoprotective mechanism by which Lut ameliorates I/R injury. In addition, the anti-oxidative activity of Lut has been confirmed by many researchers. Zhang et al. (2013) reported that Lut is the most effective of the five flavonoids isolated from Ixeris sonchifolia in regulating antioxidant activities. In a middle cerebral artery occlusion-induced ischemia rat model, 4 mg/kg Lut reduced the infarct area, protected neuronal cells from death and increased neuroprotective effects (Zhang et al., 2013). HO-1 expression was significantly up-regulated by Lut pretreatment. They also found that the neuroprotective and anti-apoptotic effects of Lut were abolished by a selective HO-1 inhibitor, tin protoporphyrin IX (Kim et al., 2017). To clarify the exact mechanisms of cardiac-protection, Sun G.-B. et al. (2012) used hydrogen peroxide (H_2_O_2_)-induced cytotoxic model in the H9c2 cell line to show that Lut provided an adaptive survival response via a mechanism that involved alleviating H_2_O_2_-derived oxidative cytotoxicity by increasing HO-1 expression, enhanced the binding affinity of Nrf2 to the ARE and preventing of apoptosis in H9c2 cells. Taken together, these results indicated the vital role of HO-1 on Lut-induced cardio-protective effects (Sun G.-B. et al., 2012).

#### Lut Improves Cardiomyocyte Function by Regulating MAPKs Family

Three members of the mitogen-activated protein kinase (MAPK) family are extracellular signal-regulated kinases (ERKs), C-Jun N terminal kinase/stress-activated protein kinases (JNK/SAPKs) and p38. Cardiomyocyte function can be regulated by MAPKs after I/R injury. Cheng et al. (2010) reported that Lut reduced the JNK, ERK and p38 protein expression to which prevented apoptosis associated neuronal death in rat primary cortical cultures. Yu et al. (2015) also found Lut inhibited the ROS-activated MAPK pathway in myocardial I/R injury. In the cardiovascular system, our research group found that the ERK/PP1a/PLB/SERCA2a and JNK pathways participated in Lut mediated cardiac-protection both *in vitro* and *in vivo* after I/R. Lut or JNK inhibitor SP600125 significantly enhanced the contraction of cardiomyocytes, improved cardiac function, reduced infarct size and decreased LDH activity. It was noteworthy that, ERK1/2 was not activated during I/R, and ERK1/2 inhibitor PD98059 had no effect on the above indices. However, Lut pretreatment concomitantly increased the phosphorylation of ERK1/2 (p-ERK1/2) and the protective effects of Lut were abrogated by PD98059 (Wu X. et al., 2013).

The p38 is another downstream effector of the MAPK pathway. It has been demonstrated that inhibition of p38 alleviated cardiomyocytes I/R injury and afforded cardiac-protection (Bogoyevitch et al., 1996; Kumphune et al., 2012). Our experiments also showed that p38 expression was increased during I/R process and that Lut decreased it. On the other hand, the p38 MAPK pathway inhibitor SB203580 and Lut inhibited the phosphorylation of PLB (*p*-PLB), hence increased the activity of SERCA2a, alleviated the calcium overload which promoted the recovery of the mitochondrial membrane potential (Δψm) and reduced the calcium overload-induced apoptosis in I/R injury (Zhu et al., 2017).

#### Lut Regulates NO and NO Synthases Isozymes to Prevent Injury from Free Radicals

The isozymes of nitric oxide (NO) synthase have been identified in humans: isozyme I (nNOS) that mainly exist in neuronal and epithelial cells, isozyme II (iNOS) that is primarily found in cytokine-induced cells, and isozyme III (eNOS) that is mainly expressed in endothelial cells. NO and NO synthases isozymes closely interact with superoxide radicals. In rats I/R injury model, the cardiac-protective capacity of Lut is partly mediated by down-regulation of NO production and its antioxidant activities. Liao et al. (2011) detected that pretreatment with Lut decreased the plasma NO levels so as to prevent NO from interacting with superoxide radicals. iNOS mRNA and protein level were down-regulated, but nNOS and eNOS expression levels were not significantly altered. On the contrary, another group found that, in diabetic patients and animals, the NO-eNOS pathway was down-regulated, and that Lut protected diabetic hearts against I/R injury by activating the myocardial eNOS pathway including the enhancing manganese superoxide dismutase (MnSOD) activity and inhibiting of mitochondrial permeability transition pore (mPTP) opening (Yang et al., 2014). Despite the fact that eNOS up-regulated MnSOD activity and increased mPTP opening, further studies are needed to elucidate the underlying regulatory mechanism.

As a major complication in the CAD treatment I/R injury, I/R injury is associated with high mortality. The evidences reviewed above indicate that Lut is a promising anti-I/R agent that impacts a wide range of proteins that are involved in the protective pathways during I/R injury. It is clear that Lut works on AKT and MAPKs to alleviate cardiomyocyte apoptosis and to improve cardiomyocyte function, respectively. Lut also blocks oxidative stress and prevents injuries arising from free radicals through the HO-1 and NO signaling pathways as illustrated in **Table 1**. The success of investigations on the mechanisms of I/R injury in CAD are based on successful modeling of I/R injury *in vivo* or sI/R *in vitro*. Previous I/R injury models in rats or mice were constructed by left anterior descending (LAD) ligation. The silk suture slipknot was used to ligate the distal 1/3 of LAD, and then the slipknot was released after 30 min of ischemia to allow for reperfusion of the myocardium. As an alternative to these classical methods, we opted for endovascular balloon occlusion (2.5 × 8 mm balloon size for mice and 3.5 × 15mm balloon size for rats) to induce I/R models. This outer balloon ligation method is more efficient and cause less damage to the tissue than conventional approaches (Hu F. et al., 2016). One limitation of the available studies on I/R models is that many studies were performed on non-cardiac cell lines such as H9_C_2 cells and HL-1 cells. These cell lines might not model cardiac cells with high fidelity and hence isolated cardiomyocytes using Langendorff system or enzyme digestion methods may be a more reliable choice to study I/R mechanism.

**Table 1:** Effect and target effectors of Luteolin in coronary artery disease.

Dose	Model	Target effectors	End points		Reference
			Increase	Decrease	
**Coronary Artery Disease**					
40 μmol/l 8 μmol/l	Landendorff – perfused rat I/R heart sI/R cardiomyocytes	AKT	*p*-Akt, *p*-PLB, SERCA2a, Bcl-2, Bcl-2/Bax ratio, cardiomyocyte shortening amplitude	Apoptotic rate, Bax, infarct size, LDH release	Fang et al., 2011
200 mg/kg	Myocardial I/R rat model	AKT	SERCA2a activity, Akt, *p-*Akt 308, and *p*-Akt 473, Bcl-2	I/R-induced myocardial infarct size, LDH release, apoptosis, Bax, cleaved caspase-3	Nai et al., 2015
10 μg/kg	Myocardial I/R diabetic rat model	AKT	p-Akt 308, and p-Akt 473, left ventricular EF, FGFR2 and LIF protein expression, p-Bad	Myocardial infarct size, LDH release, incidence of arrhythmia, apoptotic death, the ratio of Bax to Bcl-2, MPO expression, (IL-6, TNF-a and IL-1a)	Sun D. et al., 2012
160 mg/kg 87 μmol/l	ISO-induced myocardial injury model H_2_O_2_-induced cytotoxicity model in H9c2 cells	HO-1	Electrocardiography, heart vacuolation, the free radical scavenging and antioxidant potential, HO-1, binding ability of Nrf2 to antioxidant response element	Serum cardiac enzymes, apoptosis, AKT and ERK,	Sun G.-B. et al., 2012
70 mg/kg 30 μmol/l	Myocardial ischemia/reperfusion injury model H9c2 sI/R	MAPKs	Cardiac function, protect ultrastructure of cardiac muscle cells, myocardial Ultra structure, ERK1/2, Mn-SOD mRNA levels, mitochondrial membrane potential	Infarct size, LDH, CK, T-SOD, MDA release, H9c2 cell apoptosis *p*-p38 MAPK/p38 MAPK ratio*p*-JNK/JNK ratio, p47-phox, HO-1 and HSP27	Yu et al., 2015
8 μmol/l 40 μmol/l	Langendorff perfused heart Cardiomyocytes	MAPKs	Contraction of the isolated heart and cardiomyocytes, p-ERK1/2, Bcl-2, SERCA2a, *p*-PLB	Infarct size and LDH activity, apoptosis, *p*-JNK, Bax, *p*-PP1a	Wu X. et al., 2013
8 μmol/l 40 μmol/l	Simulated I/R cardiomyocytes Langendorff perfused heart	MAPKs	*p*-PLB, activity of SERCA2a, the recovery of the Δψm, Bcl-2 and caspase-3	Calcium overload, apoptosis, activation of the p38 MAPK pathway, Bax and cleaved caspase-3	Zhu et al., 2017
10 μg/kg	Myocardial IR injury model	NOs		Ventricular tachycardia and ventricular fibrillation incidence ratio mortality, myocardial infarct size, LDH release NO levels	Liao et al., 2011
100 mg/kg	Streptozotocin induced diabetic rats IR model	NOs	MnSOD, eNOS expression	LDH release, MDA, Ca^2+^-induced mPTP opening, mitochondrial inner membrane potential	Yang et al., 2014

### Lut Improves Cardiac Function in HF

**FIGURE 3:**
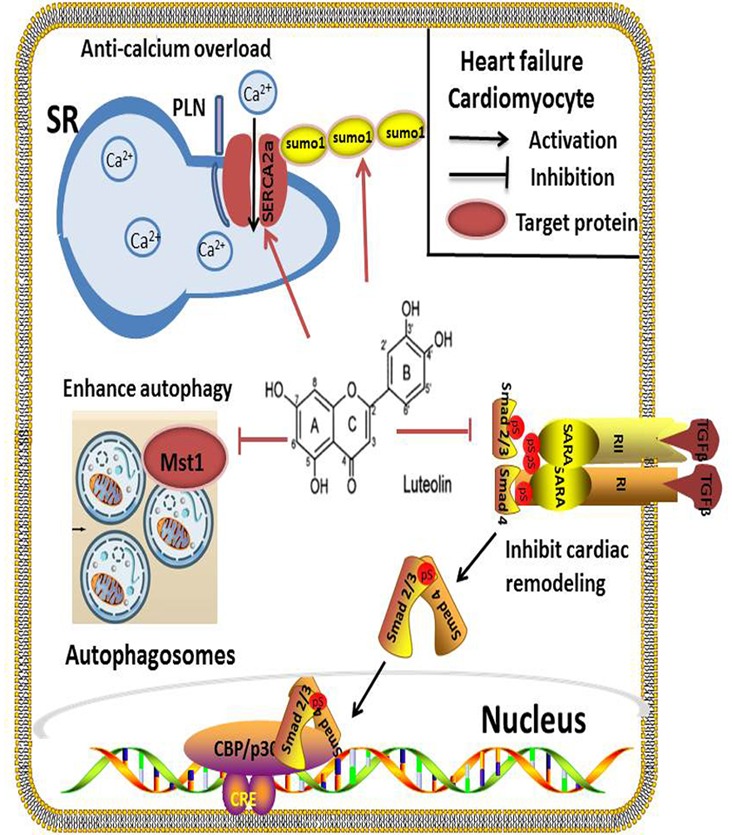
In HF, SERCA2a is a vital target regulated by Lut for effective cardiomyocyte contraction and relaxation. Inhibition of SERCA2a activity markedly abolishes Lut-induced benefits *in vitro* and *in vivo*. Autophagy is a self-protection response to stress in HF. Lut up-regulates autophagy through MST1 inhibition, alleviating post-infarction cardiac dysfunction and enhancing autophagic flux in the neonatal cardiomyocytes after hypoxia. Lut protects against cardiac fibrosis and hypertrophy by blocking the Ang II-TGFβ1 signaling pathway, and suppressing expression of TGFβ1.

Lut has not only been demonstrated to increase myocardial contractility during I/R injury, but also has received considerable attention from many researchers regarding its long-term application in HF treatment. Wang et al. (2013) reported that long-term Lut treatment of doxorubicin-induced HF in rats was able to improve the cardiac function, in part, by reversing ventricular remodeling. In diabetes mellitus-induced cardiac dysfunction, Lut attenuated myocardial oxidative stress and reversed the reduction of cardiac function (Wang et al., 2012). Studies that have explored the effectiveness and mechanisms of Lut in improving cardiac function in HF have mainly focused on its regulation of cardiomyocytes contractility, enhancement of autophagy in cardiomyocytes and limitation of cardiac remodeling (**Figure 3**).

#### Lut Improves Systolic and Diastolic Function of Cardiomyocytes through SERCA2a

Calcium (Ca^2+^) recycling is a pivotal process for effective cardiomyocyte contraction and relaxation. SERCA2a is an ATP-dependent enzyme that regulates SR Ca^2+^ stores by pumping Ca^2+^ into the SR. It is important to modulate cardiac cytosolic Ca^2+^ levels. Prior investigations have revealed that the expression and activity of SERCA2a were altered in many heart diseases, such as HF and cardiomyopathies. SERCA2a dysfunction causes Ca^2+^recycling disorders resulting in severe impairment of cardiomyocyte systolic and diastolic functions (Zsebo et al., 2014; Braunwald, 2015). SERCA2a has become a promising target of pharmacological or gene therapy in HF. Our previous work indicated that Lut improved the contractility of cardiomyocytes by modulatingSERCA2a during I/R injury. In a HF rat model, we also found that Lut improved cardiac dysfunction and significantly increased SERCA2a expression, activity and stability, which improved Ca^2+^ transient and cardiomyocyte contractility. The levels of AKT, phospholamban (PLB), and specificity protein 1 (Sp1), which are capable of upregulating SERCA2a expression and activity, were also increased in the study (Hu et al., 2017). Based on another study, small ubiquitin-related modifier (SUMO) 1, a post-translationally modified protein that can enhance SERCA2a activity is concurrently increased by Lut (Kho et al., 2011; Tilemann et al., 2013). To confirm the function of SERCA2a in Lut-mediated cardiac protection, inhibition of SERCA2a activity markedly abolished Lut-induced benefits, and inhibition of PI3K/AKT and the MAPKs pathway (ERK, p38) produced the same results (Wu X. et al., 2013; Zhu et al., 2017).

#### Lut Enhances Autophagy by MST 1 Inhibition

Autophagy is a self-process cell constituents through the lysosomal degradative pathway. This physiological process is essential for protecting cells against excessive or dysfunctional organelles and for maintaining cellular homoeostasis (Takemura et al., 2006). In HF or ischemic cardiomyopathy disease, autophagy acts as a self-protective mechanism in response to stress. Inefficient autophagy leads to poor myocardial performance (Yan et al., 2005; Gustafsson and Gottlieb, 2009). Mammalian sterile 20-like kinase 1 (Mst 1) inhibits autophagy by impairing protein quality control mechanisms in the heart cells. Hu J. et al. (2016) reported that Lut up-regulated autophagy through Mst1 inhibition which alleviated post-infarction cardiac dysfunction in the Lut pretreatment group, enhanced autophagic flux, and lessened the aggresomes accumulation in neonatal cardiomyocytes in hypoxia model. Furthermore, in Mst1^+/-^ murine model, the protective effects of Lut were abolished (Hu J. et al., 2016). Taken together, the interaction between Lut and Mst1 is a crucial pathway that regulates autophagy in cardiac cells. These findings are important as autophagy is a hallmark of many CVD. Hence, how Lut impacts autophagy at different stages awaits further investigation in order to better exploit its cardiac benefits.

#### Lut Protects against Cardiac Remodeling by Restoring Ang II-Induced Profibrotic Cytokine TGFβ1 Expression

Cardiac remodeling is a pathological process associated with HF. It is characterized by neurohormonal activation, compromised the heart’s response to a hemodynamic load and/or injury and alteration to the structure (dimensions, mass, and shape) (Cohn et al., 2000). Cardiac fibrosis and hypertrophy are two types of remodeling patterns, which impair cardiac function and metabolism over time. Oxidative stress has been confirmed as a risk factor for cardiac remodeling (Zhang et al., 2012). Anti-oxidative compounds may have protective effects against cardiac remodeling. Blockade of the Ang II-TGFβ1 signaling pathway has been demonstrated as an effective strategy to prevent cardiac fibrosis in animal models.

Lut has been reported as an anti-fibrotic compound in the lung and liver (Domitrovic´ et al., 2009; Chen et al., 2010). The renin-angiotensin system (RAS) participates in the pathological process of cardiac remodeling; Ang II is the initial factor of RAS and the pro-fibrotic activator of cardiac fibroblasts (Werner and Böhm, 2008). It stimulates pro-fibrotic cytokine transforming growth factor β (TGFβ1) expression (Rosenkranz, 2004). Nakayama et al. (2015) clearly demonstrated that in Ang II-induced hypertension rats, TGF β1, CTGF, Nox2, and Nox4 gene expression were increased and oral pretreatment with a high-luteolin diet restored the above indices, and also suppressed H_2_O_2_-induced TGFβ1 expression. Food-derived Lut may be a therapeutic strategy through the inhibition of Ang II-induced cardiac remodeling (Nakayama et al., 2015).

According to these reports, Lut appears to improve myocardial contractility by increasing the expression and function of SERCA2a, up-regulating autophagy through Mst1 inhibition, and preventing cardiac fibrosis by blocking the Ang II-TGFβ1 signaling pathway (**Table 2**). Moremore, different *in vitro* and *in vivo* models have been constructed using different Lut working concentrations; the optimal concentration of Lut should be defined by cytotoxic effect test and cell function detection. For example, in isolated ventricular myocytes, the shortening amplitude of single cardiomyocytes was detected after pretreating the myocytes with different Lut concentrations (4, 8, 16 μM). The maximum cell shortening was recorded at the concentration of 8 μM and without affecting cellular activities. This concentration of Lut is appropriate for application in isolated ventricular myocytes for HF research.

**Table 2:** Effect and target effectors of Luteolin in heart failure.

Dose	Model	Target effectors	End points		Reference
			Increase	Decrease	
**Heart Failure**					
8 μmol/lM	Abdominal aortic constriction operation SD rat model	SERCA2a	Contractility, Ca^2+^ transients, stability of SERCA2a, SUMO 1, Akt, PLB, Sp1, Bcl-2/Bax, caspase-3/cleaved-Caspase3	Myocardium fibrosis	Hu et al., 2017
40 μmol/l	Simulated I/R cardiomyocytes				
8 μmol/l	Simulated I/R cardiomyocytes	MAPKs	*p*-PLB, activity of SERCA2a, the recovery of the Δψm, Bcl-2 and caspase-3	Calcium overload, apoptosis, activation of the p38 MAPK pathway, Bax and cleaved caspase-3	Zhu et al., 2017
40 μmol/l	Langendorff perfused heart				
10 μg/kg	Myocardial infarction model	MST1	LVEF, LVFS and ‘ LV dp/dt max, Bax/Bcl-2 ratio, GFP-LC3 puncta, LC3-II and beclin1, mitochondria function of cardiomyocytes	LVEDD, LVESD, Creatine kinase-MB, LDH, IL-1a MPO and TNF-a, cardiomyocytes apoptosis, caspase-3 activity, accumulation of aggresomes, P62 Mst1, *p*-Mst1	Hu J. et al., 2016
Oral pre-administration 0.035% luteolin	Angiotensin II-induced hypertension model	Ang II		Left ventricular wall thickness, weight of left ventricular, fibrosis of the left ventricular wall, Cardiomyocyte diameter, oxidative fluorescence intensity, TGFβ1, CTGF, Nox2, Nox4, ANP, and BNP, *p*-JNK	Nakayama et al., 2015
20 μmol/l	Adult rat cardiac fibroblasts				

### Anti-Atherosclerosis (AS) Mechanism of Lut

**FIGURE 4:**
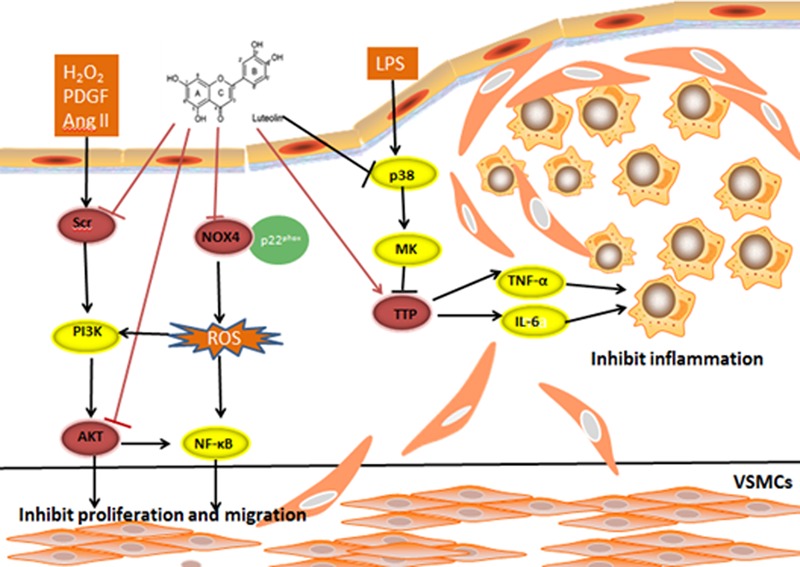
H_2_O_2_, PDGF, and AngII are stimuli for VSMC proliferation and migration. An inhibitory effect on the Ang II-induced VSMC proliferation and migration rate was observed following pretreatment with Lut. Lut decreases p-AKT (308) and p-AKT (473) expression to inhibit VSMC proliferation and migration. Lut blocks H_2_O_2_-triggered SRC phosphorylation. ATP-binding pocket may be the Lut-binding site in SRC. Lut affects the TNF-α and IL-6 mRNA stability from inhibiting the p38 and MK2 phosphorylation levels by promoting TTP expression. The mRNA stability is known to impact inflammatory processes by regulating inflammatory gene expression. Lut suppresses TNF-α-activated ROS generation, and decreases the NOX4 and p22^phox^ expression as well as attenuates oxidative stress through the NOX4/ROS-NF-κB pathways.

Atherosclerosis is a chronic inflammatory artery disorder that can affect coronary arteries, the peripheral vasculature, and cerebrovascular structures. Genetic, environmental, psychosocial and pathological factors cause may contribute to the onset of this multi-stage, multi-factorial disease. The pathogenesis of AS comprises lipid accumulation, endothelial dysfunction, cell proliferation and migration, and inflammation (Hansson, 2005). Lut has been demonstrated in many studies to have anti-inflammatory effects, and to inhibit cell proliferation and migration (Cheng et al., 2013; Kritas et al., 2013; **Figure 4**).

#### Lut Suppresses Up-Regulation of AKT to Inhibit Proliferation and Migration of VSMCs

AKT is essential for vascular smooth muscle cell (VSMC) proliferation and migration. Ablation of AKT caused severe atherosclerotic plaques (Fernandez and Ljenkins, 2009). Using MTT and transwell chamber assays, Ang II-induced VSMC proliferation and migration were inhibited, and the levels of p-AKT (308) and p-AKT (473) levels were decreased in the Lut group compared to the Ang II group. These findings strongly suggest that Lut down-regulates the AKT signaling pathway and inhibits the proliferation and migration of VSMCs (Jiang et al., 2013; Xu et al., 2015).

#### Binding of Lut at the ATP-Binding Pockets of Src Kinase Suppresses Inflammatory Response

The Src kinase signaling pathway is involved in the progression of AS in VSMC proliferation and migration. It also participates in reactive oxygen species (ROS)-induced pathophysiological processes of VSMCs. H_2_O_2_ is one type of ROS, which can elicit migration of VSMCs. Lut can block H_2_O_2_-triggered Src phosphorylation. It was shown that activation of Src in the Lut group was significantly lower compared with that of the H_2_O_2_ group. These results indicated that Lut can suppress H_2_O_2_-directed migration and proliferation in VSMCs (Lang et al., 2012). Furthermore, via blockade of Src and Syk, Lut suppressed nuclear translocation of NF-κB but did not suppress other MAPKs. In comparison with wild type and point mutant Src, Lut strongly suppressed the phosphorylation of Src, and ATP could compete with Lut at the ATP–binding pocket of Src. When the concentration of ATP was increased from 400 to 800 μg, the suppression of Src by Lut was abrogated, suggesting that the ATP-binding pocket may work as a binding site of Lut in Src (Lee et al., 2014).

#### Lut Participates in TTP-Mediated Regulation of TNF-α and IL-6 mRNA Stability to Inhibit LPS-Inflammatory Responses in Macrophages

The pathogenesis of AS is also linked to a chronic inflammatory processes. mRNA stability is known to regulate inflammatory gene expression to impact inflammatory processes (Wu and Brewer, 2012). Tristetraprolin (TTP) is regarded as a substrate of p38 MAPK-activated protein kinase 2 (MK2), which can directly target mRNA to the exosome for rapid degradation by binding to adenine-uridine-rich elements (AREs) in the 3′ UTR of target mRNAs (Lai et al., 1999; Shi et al., 2012). A previous study reported that activation of p38/MK2 pathway can abolish TTP-mediated repression of IL-6 3′-UTR reporter activity (Zhao et al., 2011). However, it remained elusive whether mRNA stability is involved in the anti-AS mechanism of Lut. Wu W.L. et al. (2013) first found that Lut, in a dose-dependent manner, suppressed the expression of” tumor necrosis factor-α (TNF-α) and interleukin-6 (IL-6) in macrophages. TTP expression was enhanced in the Lut group, but another RNA binding protein, HuR, was not significantly altered. To examine whether Lut affects TNF-α and IL-6 mRNA stability through TTP activation, we compared the Lut and LPS groups and found that by promoting TTP expression, Lut inhibited the p38 and MK2 phosphorylation level (Wu W.L. et al., 2013).

#### Lut Attenuates TNF-(α-Induced Oxidative Stress through NOX4

Oxidative stress is regarded as a contributing factor during the AS disease progression. NOX4 is widely expressed in the heart (Ago et al., 2010), and it is the major source of ROS generation (Bedard and Krause, 2007; Sumimoto, 2008). Xia et al. (2014) reported that Lut may be an effective agent for preventing AS by protecting ECs from TNF-α-induced inflammatory responses and oxidative damage. They found that Lut suppressed TNF-α-induced ROS generation, and it decreased NOX4, p22^phox^expression. In turn, down-regulated NOX4 reduced the expression of BCL-2, ICAM-1, and VCAM-1. They proposed the formation of a ternary complex involving NOX4 and ROS with NF-κB that regulated TNF-α-induced oxidative stress. This hypothesis was based on the following evidences: upon exposure to TNF-α in ECs, NF-κB inhibitor pyrrolidinedithiocarbamic acid (PDTC) inhibited Nox4 expression, while Nox inhibitor diphenyleneiodonium (DPI) deceased NF-κB activation. Moreover, the DCF-detected ROS fluorescence intensity induced by TNF-α are both attenuated by PDTC and DPI. However, the interactions between and structures of the three constituents are needed to be confirmed in the further (Xia et al., 2014).

Anti-atherosclerosis properties of Lut are shown in **Table 3**. Lut inhibits the proliferation and migration of VSMCs by regulating AKT and Src, and decreases inflammation and oxidative damage through TTP activation related to mRNA stability and NOX4/ROS-NF-κB pathways. Inflammation and oxidative stress are the main accelerating factors of AS. As the capacities of Lut may be applied to prevent cardiovascular system from AS formation, especially in the early stage of AS or unstable plaques, well-designed studies are needed to explore therapeutic effects of Lut in different stages of AS and different types of plaques.

**Table 3:** Effect and target effectors of Luteolin in atherosclerosis.

Dose	Model	Target effectors	End points		Reference
			Increase	Decrease	
**Atherosclerosis**					
50 μmol/l	Vascular smooth muscle cells	AKT		*p*-Akt (308), *p*-Akt (473), PCNA	Xu et al., 2015
50 μmol/l	Ang II-stimulated smooth muscle cells	AKT		SMC proliferation	Ding et al., 2014
				SMC migration VEGF, NOX4, *p*-Akt	
50 μmol/l	Vascular smooth muscle cells	SRC		proliferation and, migration *p*-Src, *p*-PDK1, *p*-Akt(308), *p*-Akt(473)	Lang et al., 2012
50 μmol/l	Lipopolysaccharide induced inflammatory responses in bone marrow macrophages	TTP	TTP	TNF-α, IL-6, *p*-P38, *p*-MK2	Wu W.L. et al., 2013
25 μmol/l	Human umbilical vein endothelial cells	NOX4	SOD, Intracellular GSH, Bcl-2,IκB-α	LDH, ROS, Nox4, p22^phox^, Caspase-3, Caspase-9, ICAM-1, VCAM-1, p-NF-κB/p65, *p*-p38, *p*-ERK1/2	Xia et al., 2014

## Conclusion and Perspective

A number of current studies seek to explore ways to reduce the incidence of CVD and improve the therapeutic effectiveness. This review summarizes the molecular mechanisms and cardiovascular effects of Lut and its role in HF, I/R injury, and AS. Lut alleviates cardiomyocyte apoptosis and blocks oxidative stress, all of which promote cytoprotection and reduce I/R injury. The data sets reviewed also provide evidence that Lut improves heart function by enhancing contractility, up-regulating autophagy, and preventing cardiac fibrosis. The anti-atherosclerosis properties of Lut stems from inhibiting the proliferation and migration of VSMCs, decreasing inflammation and ameliorating oxidative damage. Overall, Lut, which is a ubiquitous dietary flavonoid, is efficacious in cardiovascular protection, and shows promise as a preventive and treatment option for CVD.

Although multiple mechanisms of the protective function of Lut on the heart have been addressed, limitations exist in the following aspects. First, previous studies mainly focused on basic research. Therefore, we lack large clinical trials designed to explore the clinical utilization of Lut, especially on the effective therapeutic dosage, and the safety of long-term administration. Based on the evidences of dosages in observational clinical trials or experimental animal studies, the human dietary intake of Lut appears to be much smaller than that used for rodent experimental studies, this result implies that intake of Lut from food may work as a preventative method for CVD, while this intake is insufficient to be an treatment dosage of CVD. Therefore, well-designed prospective clinical trials to observe the effective therapeutic dosages of Lut on patients with myocardium I/R injury, HF, and AS would greatly advance knowledge on its clinical application. In an open-label pilot study, Lut effectively reduced autism spectrum disorders symptoms on behavior in children, which was based on the antioxidant, anti-inflammatory, and neuroprotective effects of Lut. Major adverse effects had not been observed in children who took 10 mg/kg of Lut (ClinicalTrials.gov identifier: NCT01847521) (Taliou et al., 2013). Although it is not a cardiovascular clinical research, 10mg/kg may be used as a reference dosage of safety in future CVD trials. Second, in-depth studies of mechanism need to focus on the synthetic function of Lut on its target effectors and signaling pathways. Further *in vitro* and *in vivo* studies should aim to identify the relationship and interaction of these effectors. We hope this review generates the scientific awareness and interest in Lut and provokes that further research by scientists from different fields would boost understanding of the molecular functions of Lut. In conclusion, extensive studies in this area are needed, and appreciation of the molecular mechanisms of Lut may pave the way for development of Lut-based therapeutic agents for CVD.

## Author Contributions

DL and YL outlined the review. YL drafted the manuscript. YL and PS edited and reviewed the manuscript. DL, YL, and PS re-reviewed it and all authors approved it.

## Conflict of Interest Statement

The authors declare that the research was conducted in the absence of any commercial or financial relationships that could be construed as a potential conflict of interest.
